# A RAD-based linkage map and comparative genomics in the gudgeons (genus *Gnathopogon*, Cyprinidae)

**DOI:** 10.1186/1471-2164-14-32

**Published:** 2013-01-16

**Authors:** Ryo Kakioka, Tomoyuki Kokita, Hiroki Kumada, Katsutoshi Watanabe, Noboru Okuda

**Affiliations:** 1 Department of Zoology, Graduate School of Science, Kyoto University, Kitashirakawa-Oiwake-cho, Sakyo-ku, 606-8502, Kyoto, Japan; 2 Department of Marine Bioscience, Fukui Prefectural University, 1-1 Gakuen-cho, 917-0003, Obama, Japan; 3 Center for Ecological Research, Kyoto University, 509-3, Hirano 2-chome, 520-2113, Otsu, Japan

## Abstract

**Background:**

The construction of linkage maps is a first step in exploring the genetic basis for adaptive phenotypic divergence in closely related species by quantitative trait locus (QTL) analysis. Linkage maps are also useful for comparative genomics in non-model organisms. Advances in genomics technologies make it more feasible than ever to study the genetics of adaptation in natural populations. Restriction-site associated DNA (RAD) sequencing in next-generation sequencers facilitates the development of many genetic markers and genotyping. We aimed to construct a linkage map of the gudgeons of the genus *Gnathopogon* (Cyprinidae) for comparative genomics with the zebrafish *Danio rerio* (a member of the same family as gudgeons) and for the future QTL analysis of the genetic architecture underlying adaptive phenotypic evolution of *Gnathopogon*.

**Results:**

We constructed the first genetic linkage map of *Gnathopogon* using a 198 F_2_ interspecific cross between two closely related species in Japan: river-dwelling *Gnathopogon elongatus* and lake-dwelling *Gnathopogon caerulescens*. Based on 1,622 RAD-tag markers, a linkage map spanning 1,390.9 cM with 25 linkage groups and an average marker interval of 0.87 cM was constructed. We also identified a region involving female-specific transmission ratio distortion (TRD). Synteny and collinearity were extensively conserved between *Gnathopogon* and zebrafish.

**Conclusions:**

The dense SNP-based linkage map presented here provides a basis for future QTL analysis. It will also be useful for transferring genomic information from a “traditional” model fish species, zebrafish, to screen candidate genes underlying ecologically important traits of the gudgeons.

## Background

Ecological and phenotypic diversification in closely related species (or populations) provides an excellent opportunity for testing the role of natural selection in evolution [1]. Understanding the genetic architecture underlying such diversification is currently a fundamental topic in evolutionary ecology. Among the forward-genetic approaches that are currently available to study natural populations, quantitative trait locus (QTL) mapping is a useful method. QTL mapping can be used to find the genetic basis of fitness-related traits of modest heritability, if the subject species can be bred and selected for the divergent phenotypes under controlled conditions [2]. Construction of a linkage map provides an essential basis for identifying chromosomal regions containing Mendelian single-gene traits and quantitative traits by genetic linkage analysis [2]. Linkage maps also serve as a link to the genomic information of model species and related non-model species by enabling genomic comparison, thus facilitate the discovery of candidate genes of non-model organisms [3-5].

Next-generation sequencers, or massively parallel sequencers, are making it more feasible to develop a large number of genetic markers, construct highly dense linkage maps, and practice comparative genomics. Thus, advances in genomics technologies make it more feasible than ever to explore the genetic basis of adaptation in both ecological model and non-model species [6]. In particular, restriction-site associated DNA (RAD) sequencing (RAD-seq) is readily available for non-model organisms [7-9]. RAD-seq aims to explore single-nucleotide polymorphisms (SNPs) adjacent to restriction endonuclease sites. Through the massively parallel sequencing of DNA fragments flanking the restriction sites, or RAD tags, RAD-seq identifies SNPs and scores them as co-dominant markers. Hundreds or thousands of RAD-tag markers are obtained, and the markers are genotyped simultaneously for multiple individuals. RAD-seq has been successfully applied to various organisms for the construction of linkage maps [10,11], QTL analysis [12,13], linkage disequilibrium analysis [14], comparative genomics [11], and genome assembly [15].

Lake fishes provide well-known examples of adaptive radiations as well as less species-rich but still illustrative examples of adaptive divergences [16]. The environmental distinctness of lakes as compared to rivers often drives adaptive evolutionary changes in lake colonisers’ traits related to foraging and locomotion. Therefore, the lake ecosystem is a model system for examining the genetics of ecological and phenotypic diversification. Gudgeons of the genus *Gnathopogon* (Cyprinidae) are temperate freshwater fishes widely distributed in East Asia that live mostly in rivers and are associated with a benthivorous feeding style [12]. However, *Gnathopogon caerulescens* inhabits the ancient Lake Biwa in Japan, which harbours some endemic fishes that have evolved to adapt to its limnetic environment [17], showing a planktivorous feeding style [18,19]. This species markedly differs from river-dwelling *Gnathopogon* species in morphology, such as body depth, gill raker density, and barbel length. These differences are considered an evolutionary consequence of adaptive divergence caused by the divergent habitat and resource use [20]. Other limnetic *Gnathopogon* populations in several lakes show signs of parallel adaptive evolution within this genus [20,21]. However, the genetic basis for the phenotypic differentiation and parallelism remains completely unknown.

The aim of our present study is to construct a linkage map of *Gnathopogon* for comparative genomics, and for the future QTL analysis to elucidate the genetic basis of the morphological evolution of *Gnathopogon* in relation to adaptations to lake environments. We constructed an F_2_ interspecific cross between *G. caerulescens* and river-dwelling *Gnathopogon elongatus*. Using this pedigree, we constructed a linkage map using RAD-seq and searched for alleles with unusual segregation ratios in the progeny. We also tested for synteny and gene order with other model fishes, especially the “traditional” model species, zebrafish, which belongs to the same family as gudgeons.

## Methods

### Study organisms and mapping family

*Gnathopogon elongatus* is widely distributed in western to central Japan. It lives in rivers and ponds, feeding on zoobenthos and benthic algae [18,19]. *Gnathopogon caerulescens* is a relative of *G. elongatus*[21] endemic to Lake Biwa in Japan and feeds on zooplankton exclusively in pelagic waters [18,19]. These two species can be bred easily by artificial insemination [19]. A female *G. elongatus* collected from a small inlet of Lake Biwa, Shiga Prefecture, and a male *G. caerulescens* from Iba-naiko Lagoon (connected to Lake Biwa) were used as founders of an F_2_ intercross. A single F_1_ female and male sibling were then crossed once to generate one full-sib F_2_ family that was reared under controlled conditions.

A total of 198 full-sib F_2_ progeny were sampled to construct a linkage map. Fin clips and muscle tissues were preserved in 99% ethanol at room temperature for several months for DNA samples. Following the collection of DNA samples, fish were fixed in 10% formalin. To determine sex, fixed fish were dissected, and their gonads were observed under a microscope.

### DNA extraction and RAD library construction

Genomic DNA of founders and their F_2_ progeny was extracted from the preserved samples using a DNeasy Blood & Tissue Kit (Qiagen). The concentration of extracted DNA was determined using a spectrophotometer. DNA quality was analysed using agarose gel electrophoresis. Approximately 1 μg of purified DNA was processed to obtain four RAD libraries each including 50 individuals. We followed the protocol of Etter [22] (see also [7]) and the instructions of the reagent manufacturers. In brief, genomic DNA from each individual was digested with the restriction endonuclease *Sbf*I (High fidelity; New England Biolabs). Modified Illumina adapters containing five nucleotides of barcode sequence (P1 adapters) unique to an individual in the library were ligated with T4 DNA ligase (New England Biolabs) to multiplex samples. The ligated DNA samples were pooled and sheared using a Covaris S-Series ultrasonicator (Covaris) into an average size of 500 bp. The sheared samples were size-selected to isolate DNA fragments spanning 300–500 bp by agarose gel electrophoresis. A Quick Blunting Kit (New England Biolabs) was used to convert 5^′^ or 3^′^ overhangs into phosphorylated blunt ends, and Klenow fragment (exo^-^; New England Biolabs) was then used to add adenine to the 3^′ ^end. An adapter with divergent ends (P2 adapter) was ligated to enable selective PCR. The samples were amplified using Phusion High-Fidelity PCR Master Mix with HF Buffer (Finnzymes) by 18 cycles of PCR, and the libraries were finally purified with a MinElute column (Qiagen) to obtain approximately 20 μl (12.3–31.3 ng/μl) of sequencing libraries. PCR to the final purification was conducted twice for each pooled sample to create two sets of four libraries. The obtained RAD libraries were sequenced on an Illumina Genome Analyzer (GA) IIx in 75-bp single reads and the Illumina HiSeq 2000 in 100-bp single reads, each in four lanes of flow cells. The sequence dataset for this study was submitted to the Sequence Read Archive under accession number DRA000602.

### Genotyping

Raw Illumina reads were filtered to discard those of low quality. Sequences with ambiguous barcode sequences were also eliminated from the subsequent marker processing using Stacks ver. 0.998 [23]. Sequences were first sorted to individuals according to the barcode sequences. Sequences from the Illumina HiSeq 2000 were truncated and analysed together with those from the Illumina GAIIx to increase read depth and overcome the PCR errors and biases in the sampling across alleles, loci, and individuals that are associated with next-generation sequencing [24]. To infer RAD loci, a minimum stack depth of 3 was required to create a stack, a maximum sequence mismatch of 2 was allowed between stacks to merge into a locus within an individual, and a maximum sequence difference of 3 was allowed to infer a homologous locus between parents. Genotypes were determined at the inferred RAD loci, requiring minimum stack depth of 20 to be called as homozygous, and correcting for the neglected heterozygote alleles due to their low coverage depth.

### Linkage map construction

A linkage map was created using JoinMap 4.0 [25] for F_2_-type markers genotyped more than 85% of progeny. Markers showing significant segregation distortion (*χ*^2^ test, *P* < 0.001, d.f. = 2) were excluded. Linkage groups were identified with an independence LOD threshold of 7. Unlinked markers and small linkage groups including less than 3 markers were excluded from further analysis. The linkage map was built using the regression mapping algorithm, a recombination frequency smaller than 0.4, and a LOD larger than 1. Up to three rounds of marker positioning were conducted with a jump threshold of 5. A ripple was performed after the addition of each new marker. Map distances were calculated using Kosambi’s mapping function. Following the initial mapping, potential errors that appear as doubtful double-recombinants were identified using genotype probabilities function of JoinMap (*P* < 0.001). The suspicious genotype was replaced by a missing value as suggested by Isidore *et al.*[26] and van Os *et al.*[27]. Then, a linkage map was constructed again using the corrected dataset. Potential error elimination and linkage map construction was iterated until no dubious genotype was identified, removing markers with >20% missing value or that is distorted (*χ*^2^ test, *P* < 0.001, d.f. = 2) in each iteration. The resultant linkage maps were drawn using MapChart ver. 2.2 [28]. We also estimated the corrected length of the linkage map by multiplying the length of each linkage group by (*m* + 1) / (*m* − 1), where *m* is the number of markers in the linkage group [29]. The coverage of the genome by the linkage map was next estimated by calculating *c* = 1 − *e*^−2*dn*/*L*^, where *d* is the average interval of markers, *n* is the number of markers, and *L* is the length of the linkage map [30].

### Analysis of transmission ratio distortion

Technical artifacts may be responsible for the distorted markers, but biological processes known as transmission ratio distortion (TRD) also cause a deviation from Mendelian segregation [31,32]. First, to explore TRD, a linkage map was constructed without excluding distorted markers. Then, we compared linkage maps with and without distorted markers to find linkage groups with extensive differences in marker assignment. The comparison revealed a substantial difference in marker assignment in LG3 (see Results). Therefore, we further studied this linkage group regarding sex-specific TRD, which could be distinguished from segregation distortion due to artifacts. We sorted F_2_ progeny by sex and constructed a linkage map for each group (LG3M for male progeny; homologous linkage group was not identified for female progeny due to extensive distortion) with the same condition as above, except the LOD threshold of 10 was used for clustering markers. Segregation of markers present on LG3M was *χ*^2^-tested (*α* = 0.001, d.f. = 2) for the expected 1:2:1 segregation ratio, and the genotypic ratios of male and female progeny were plotted along LG3M to visualise the direct cause of the distortion.

### Sequence comparison

Consensus sequences of the mapped RAD-tag markers (70 bases in length) were aligned with genomic sequences of four model fishes. The zebrafish *Danio rerio* (Zv9), three-spined stickleback *Gasterosteus aculeatus* (ver. 1.0), medaka *Oryzias latipes* (ver. 1.0), and fugu *Takifugu rubripes* (ver. 5.0) genome sequences were downloaded, and blastn (BLAST+ ver. 2.2.26 [33]) searches with an *e*-value cutoff of 10^-10^ were conducted. In cases where the search of a query sequence hit two or more loci, a hit with the smallest *e*-value was considered significant; if the difference of the *e*-values between the first and the second smallest hits was not greater than 10^3^, the hit was considered insignificant. Significant hits on the chromosomes were used, including unoriented scaffolds assigned to chromosomes in the fugu genome. The Oxford grids [34] were constructed to study synteny and to compare positions of the homologous loci using Grid Map ver. 3.0a (http://cbr.jic.ac.uk/dicks/software/Grid_Map/).

## Results

### RAD-tag sequencing and genotyping

Illumina sequencing with GAIIx yielded a total of 142,563,874 75-base reads, and HiSeq 2000 sequencing yielded 697,482,400 100-base reads. The average count of RAD tags per individual was 3,842,573 (SD 966,921). RAD tags were aligned and clustered into 44,109 stacks (Additional file 1), and 11,463 candidate RAD loci were inferred. For the analysis of the F_2_ mapping population, 2,819 RAD-tag markers were informative and were scored for sufficient numbers of progeny. Among them, 1,887 markers were retained after discarding those with a deviation from a Mendelian segregation pattern, and then they were passed forward into the linkage map construction.

### Linkage map

**Figure 1:**
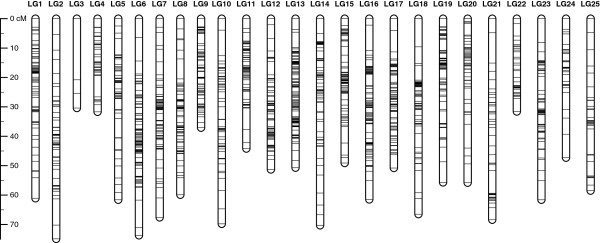
**A linkage map of the interspecific cross between *Gnathopogon caerulescens *and *Gnathopogon elongatus *.** The bars on each linkage group represent mapped RAD-tag markers. The lengths of the linkage groups are based on Kosambi cM. A detailed map is presented in Additional file 2: Figure S1.

Linkage analysis identified 25 linkage groups (LG1–LG25) containing a total of 1,622 markers (Table 1) after the removal of dubious genotypes. The sex-averaged map spanned 1,390.9 cM, with a mean distance between markers of 0.87 cM (Figure 1 and Additional file 2: Figure S1). The lengths of linkage groups ranged from 30.4 (LG3) to 74.9 cM (LG2); the number of markers mapped on a linkage group ranged from 4 (LG3) to 116 (LG13). The corrected length of the linkage map was estimated at 1,455.1 cM, which is converted to a genome coverage of 86.9%.

**Table 1:** **Summary of the sex-averaged map of*Gnathopogon***

	**No. of markers**	**Length (cM)**	**Average marker interval (cM)**
LG1	73	61.05	0.85
LG2	72	74.87	1.05
LG3	4	30.36	10.12
LG4	42	31.72	0.77
LG5	58	61.59	1.08
LG6	100	73.70	0.74
LG7	69	67.64	0.99
LG8	79	59.86	0.77
LG9	67	37.06	0.56
LG10	50	69.77	1.42
LG11	69	44.19	0.65
LG12	76	51.35	0.68
LG13	116	50.70	0.44
LG14	73	70.26	0.98
LG15	77	49.10	0.65
LG16	98	61.45	0.63
LG17	71	50.84	0.73
LG18	68	66.51	0.99
LG19	74	55.67	0.76
LG20	59	55.81	0.96
LG21	50	68.35	1.39
LG22	40	31.62	0.81
LG23	64	61.65	0.98
LG24	41	47.30	1.18
LG25	32	58.46	1.89
Average	64.88	55.64	
Total	1884	1,858.36	0.87

### Transmission ratio distortion in female progeny

**Figure 2:**
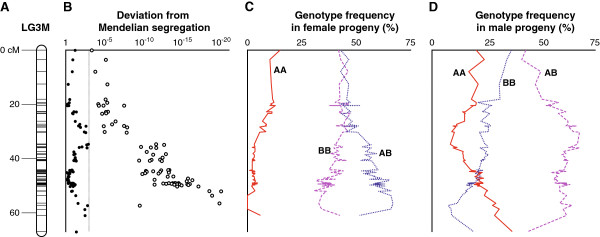
**Linkage map, extent of deviation from Mendelian segregation, and genotype frequencies in LG3M. (A)** Schematic chart of LG3M. **(B)** Degree of deviation from the expected 1:2:1 segregation along LG3M. The *x*-axis represents the position in LG3M; *y*-axis represents *χ*^2^-test *P*-values for distorted segregation. Each white dot represents female progeny; the black dots represent male progeny. The *y*-coordinate of female progeny is based on the homologous marker of the male linkage map. The dotted line represents *α* = 0.001. **(C–D)** Each line represents the genotype frequency of loci positioned on LG3M. AA denotes a homozygote derived from the grandmother; BB denotes a homozygote derived from the grandfather; AB denotes a heterozygote.

A total of 2,627 markers, including distorted ones, were assigned to 25 linkage groups. Each of them had homologous relation to one of the linkage groups identified without distorted markers. LG3D, the homologous linkage group of LG3, showed apparent differences in marker assignment: only 4 markers were mapped on LG3, whereas 120 markers were mapped on LG3D (Additional file 3: Figure S2). This was considered to be due to TRD on LG3; we therefore constructed a linkage map for LG3M to further study TRD on LG3. LG3 contained four markers over 30.4 cM; LG3M contained 81 markers over 66.9 cM (Additional file 3: Figure S2; Figure 2A).

Many of the markers assigned to LG3M genotyped in male progeny yielded larger *χ*^2^-test *P* values for the expected Mendelian 1:2:1 segregation in the F_2_ intercross than the threshold applied (*α* = 0.001, d.f. = 2), with a median of 0.11 (range 1.1×10^-3^–6.7×10^-1^; Figure 2B). In contrast, most of the same markers genotyped in female progeny yielded smaller *χ*^2^-test *P* values, with a median of 8.2×10^-13^ (range 8.9×10^-21^–4.0×10^-4^; Figure 2B), and only one marker exceeded the threshold. There was a trend for the female *χ*^2^-test *P* value to decrease toward one end of the hypothetical homologous linkage group. TRD in female progeny was mainly due to the lack of homozygote alleles derived from the grandmother (Figure 2C). Such trend was not apparent for male progeny (Figure 2D).

### Syntenic relationship between *Gnathopogon* and model fish species

**Figure 3:**
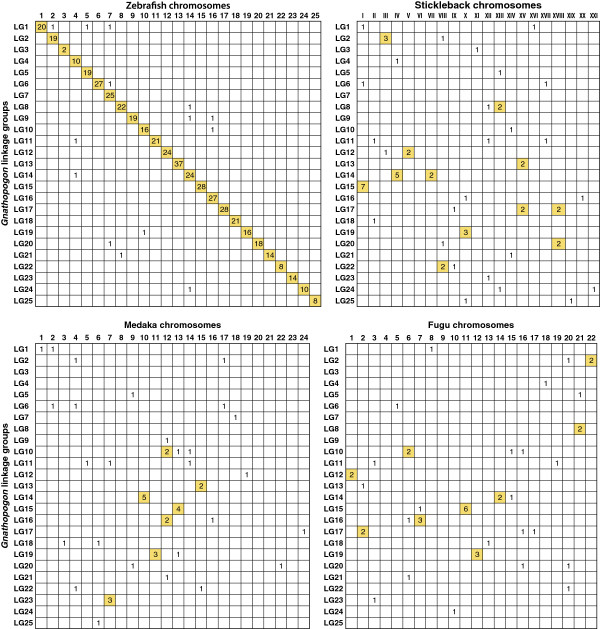
**Oxford grid comparing genomes of *Gnathopogon *and four model fishes.** Each number in a cell denotes the number of homologous pair of loci in each genome. The homologous loci were inferred from sequence similarity searches of mapped RAD-tag marker against the genome sequences of model fishes. Cells with more than one pair are highlighted in yellow.

BLAST searches of the 1,622 mapped *Gnathopogon* RAD-tag marker consensus sequences against the genome sequences of zebrafish, stickleback, medaka, and fugu indicated variation in the syntenic relationship between *Gnathopogon* and the respective species. Homology was most frequently inferred to the zebrafish genome, with 30.3% of *Gnathopogon* sequences being mapped to it. In contrast, the other three species yielded limited numbers of similarity hits. Only 3.7%, 2.8%, and 2.8% of *Gnathopogon* sequences mapped to the stickleback, medaka, and fugu genome sequences, respectively. Of the hits against the zebrafish genome, 97.1% aligned to the chromosomes that had a one-to-one relationship with *Gnathopogon* linkage groups, suggesting highly conserved synteny between *Gnathopogon* and zebrafish (Figure 3). Because of the small number of significant hits, synteny between *Gnathopogon* and the three fish species was inconclusive.

**Figure 4:**
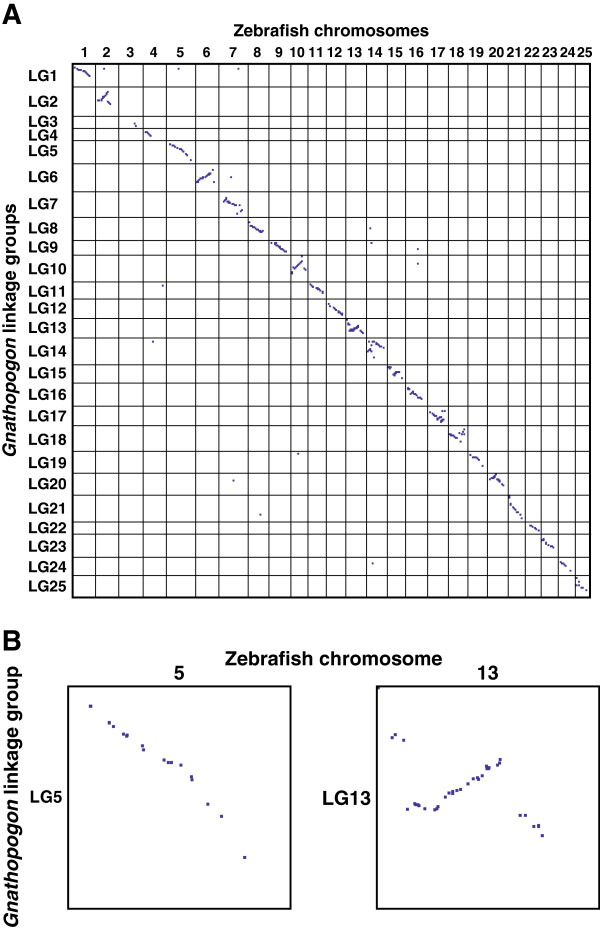
**Oxford grid comparing genomes of *Gnathopogon *and zebrafish.** Each dot represents the position of a homologous locus. The *x*-axis is proportional to physical length; the *y*-axis is proportional to Kosambi cM. **(A)** Genome-wide comparison of the positions of homologous loci. **(B)** Enlarged cells of the Oxford grid, showing extensive and disrupted collinearity of the syntenic chromosomes of *Gnathopogon* and zebrafish species.

Thirteen syntenic pairs of linkage groups and chromosomes (LG/chromosome 1, 4, 5, 6, 9, 11, 12, 19, 21, 22, 23, 24, and 25) showed apparent collinear relationships between *Gnathopogon* and zebrafish (Figure 4 and Additional file 4: Figure S3), whereas other pairs exhibited disrupted collinearity, suggesting intrachromosomal rearrangements (Figure 4B and Additional file 4: Figure S3). Thus, in addition to synteny, gene order within a syntenic chromosome was also inferred to be substantially retained between *Gnathopogon* and zebrafish.

**Figure 5:**
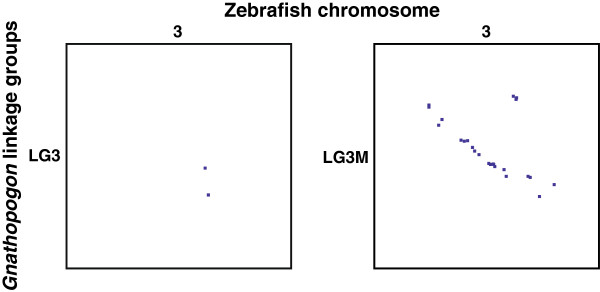
**Oxford grid comparing *Gnathopogon *linkage groups LG3 and LG3M and zebrafish chromosome 3.** Each dot represents the position of a homologous locus. The *x*-axis is proportional to physical length; the *y*-axis is proportional to Kosambi cM.

Although only two of four markers on LG3 were aligned to the zebrafish chromosome 3, 24 of 25 markers with significant hits on LG3M showed a synteny of LG3M with zebrafish chromosome 3 and collinearity of loci (Figure 5). This result further supports not only a syntenic relationship between LG3 and zebrafish chromosome 3 but also a successful mapping of LG3M, which seems to have experienced a limited influence of TRD.

## Discussion

### RAD sequencing and linkage mapping

Here, we present the first linkage map of *Gnathopogon*, which is also the first for the Gobioninae, a diverse group of fishes within the family Cyprinidae. Taking advantage of massively parallel sequencers, we obtained a high-density linkage map with 25 linkage groups and an average marker distance of approximately 0.87 cM that covers 86.9% of the genome. The number of identified linkage groups is congruent with the karyotypes of *G. caerulescens* (2*n* = 50) and *G. elongatus* (2*n* = 50) [35]. Such a dense linkage map contains detailed information on the genomic structure of an organism and is therefore useful for studies involving comparative genomics and QTL mapping.

To date, AFLP and microsatellite markers have been popular options for linkage analyses in organisms without genomic information. Although AFLP markers require no prior information about the genome of a target species, they are anonymous dominant markers bearing no sequence information for genomic comparison; microsatellite markers are sequence-based, but they are costly and time-consuming if hundreds or thousands of markers are involved. Our linkage map is solely based on RAD-seq. In contrast to AFLP and microsatellite markers, RAD-tag markers have advantages for the genomic analysis of non-model organisms. These markers are sequence-based, allowing the practice of comparative genomics [10,11], which aids in exploring candidate genes for traits of interest [13] and even assembling *de novo* genomic sequences [15]. Moreover, allelic information on a large number of markers is readily available without prior curation and labourious experiments. The present study further demonstrates the utility of RAD-seq in the genomic study of a non-model organism, yielding a wealth of genomic information without prior knowledge of the genome of a subject species.

### Sex-specific TRD

We found a female-specific TRD in the alleles of marker loci homologous to LG3M. TRD refers to a phenomenon in which the alleles of a locus of a heterozygous parent are not transmitted equally, resulting in deviation from the Mendelian 1:1 segregation [32,36]. This phenomenon is an extension of segregation distortion, referring to the unequal segregation during meiosis; TRD also includes cases in which postmeiotic effects or unknown causes yield distorted transmission of alleles. The extent of TRD is also correlated with genomic divergence, which is empirically shown as the abundance of distorted markers in interspecific crosses relative to intraspecific crosses [37-39]. The divergence time of the two *Gnathopogon* species used to construct the mapping family is estimated at 4 million years ago (mya) [21]. This might have caused substantial differences between the genomes of the two species, due to the accumulated genomic changes following the divergence. Taken together, the genomic data suggest that TRD occurred in the interspecific cross of the *Gnathopogon* lineages. To test this hypothesis, intraspecific crosses of *Gnathopogon* species would be needed.

Sex-specific TRD occurs in several animals [40-42]. A study of female-specific TRD in the mouse *Mus musculus* suggested that the TRD was caused by the post-fertilisation reduction of female viability that involved a specific region of a chromosome [40]. The female-specific TRD in *Gnathopogon* also seems to be due to postzygotic causes, such as the reduced viability of the female embryo or fry involving a deleterious gene on LG3 or a deleterious gene regulated by a gene on LG3. This gene might be a recessive lethal allele derived from the female founder, *G. elongatus*. Male viability might not be reduced because the lethality of hybrids is rescued by a gene in the male-determining region. This explanation seems likely because male offspring exhibited no such TRD on LG3M and because the allele frequency in female progeny exhibited the trend along LG3M. Further studies are needed to elucidate the mechanism of TRD by investigating the survivability of gametes and zygotes and the allele transmission using interspecific and intraspecific crosses.

TRD can affect the transmission of alleles in the hybrid zone. In mapping populations of the iris *Iris fulva* and *Iris brevicaulis*, for example, TRD causes an asymmetric introgression of alleles of *I. fulva*[43], which is attributable to the more frequent introgression of *I. fulva* alleles into *I. brevicaulis* in the natural hybrid zones between the iris species [44]. Our subject species, *G. caerulescens* and *G. elongatus*, show parapatric distribution in the Lake Biwa basin. *G. elongatus* inhabits the tributaries, lagoon, and shallow littoral zone of the lake. Conversely, *G. caerulescens* inhabits the offshore limnetic zone. However, *G. caerulescens* spawns in the lagoon and littoral zone, and the reproductive seasons of these species overlap, resulting in reproductive season sympatry the coexistence during the reproductive season [19]. These species occasionally hybridise in natural habitats, that is, the premating barrier is incomplete ( [19], Kokita, unpublished data). The TRD might contribute to reproductive barriers between sympatric *Gnathopogon* species by lowering the fitness of hybrids because hybrid individuals produce a smaller number of viable offspring [45].

### Genomic comparison

There was high synteny between *Gnathopogon* and zebrafish. Majority of the RAD loci located on a *Gnathopogon* linkage group are colocalised to a single zebrafish chromosome. Considering the old divergence of the lineages leading to each species, which date back to 117 mya (95% CI, 100–135 mya) [46], this is a substantial conservation. It is therefore likely that extensive interchromosomal rearrangements have not occurred in either of the lineages leading to *Gnathopogon* and zebrafish since they diverged. This conclusion supports the findings from the comparative analysis of genomic structure among fish and mammalian species indicate that interchromosomal rearrangements are less frequent in teleost fishes than in mammals [47-50]. Collinearity was also general between *Gnathopogon* and zebrafish, yet interruptions of collinearity were not rare. These data suggest that intrachromosomal rearrangements, such as inversions, occurred in either or both of the two lineages after the divergence of their ancestors.

Lineages including *Gnathopogon*, zebrafish, or the common carp *Cyprinus carpio* are major lineages within Cyprinidae that diverged in the early stage of the diversification of cyprinid fishes [46,51]. Cyprinid fishes show great cytogenetic variation. Their chromosome numbers range from 2*n* = 42 (*Acheilognathus gracilis*[52], or 2*n* = 30 if the taxonomically controversial *Paedocypris carbunculus* is placed within Cyprinidae [53]) to 2*n* = 417–470 (*Ptychobarbus dipogon*[54,55]), with a mode at 2*n* = 50, followed by 2*n* = 48 [56]. Thus, it has been suggested that the ancestral karyotype of cyprinid fishes was 2*n* = 48–50, and that polyploidisation occurred in several groups within the Cyprinidae [57-63]. Genome-scale syntenic analyses between zebrafish and other cyprinid fishes have been conducted for common carp (2*n* = 100) [64] and grass carp *Ctenopharyngodon idella* (2*n* = 48) [65], both of which revealed some cases of interchromosomal rearrangements. The majority of the linkage groups in common carp have two-to-one relationships with zebrafish chromosomes, suggesting tetraploidisation in the common carp lineage. Those analyses also revealed a common carp linkage group sharing loci with two zebrafish chromosomes, which is speculated to have resulted from a chromosome recombination or transposition followed by fusion of homologous chromosomes during the process of diploidisation following tetraploidisation [64]. Most grass carp linkage groups are syntenic with zebrafish chromosomes, many of which have one-to-one relationships. One grass carp linkage group exhibits a one-to-two relationship with zebrafish chromosomes, suggesting chromosomal fusion. In grass carp, substantial macrosynteny and several cases of interchromosomal rearrangements are suggested. Considering the macrosynteny between *Gnathopogon* and zebrafish and the substantially straightforward trace of autopolyploidisation in the genome of common carp, the ancestral karyotype of Cyprinidae seems to be 2*n* = 50, concordant with the inference from the comparative karyological studies [62,63]. On the other hand, syntenic analysis between zebrafish and Mexican cave tetra *Astyanax mexicanus* (Characidae; 2*n* = 50) revealed cases of putative interchromosomal rearrangements [3], such that syntenic loci of an *Astyanax* linkage group resided on several zebrafish chromosomes. These were suggested to be caused by gene duplications after the divergence of the lineages 248 mya (95% CI, 227–268 mya) [66]. Nevertheless, the analysis also revealed that synteny was conserved between *Astyanax* and zebrafish in numerous genomic regions. Combining the syntenic relationships and the genomic information of zebrafish, candidate genes for ecologically and evolutionarily important traits were identified in *Astyanax*[3].

Cyprinidae is the largest family of freshwater fishes. They have highly diverse morphology, ecology, and physiology, which are adapted to the vast range of habitats and resources they exploit [55,67]. Evolutionary ecological studies have been conducted in various cyprinid species concerning, *e.g.*, adaptive radiation [68], hybridisation [69,70], and resource polymorphism [71]. However, the genomic basis and consequences of their diversification have not been extensively explored. In this study, *Gnathopogon* are suggested to be able to take advantage of the genomic information of a model cyprinid species, zebrafish, and its conserved synteny and collinearity with *Gnathopogon*. They may provide a prediction of candidate genes responsible for the traits related to phenotypic divergence that have ecological and evolutionary significance. Conservation of synteny and collinearity might be expected among cyprinid fishes, which could be advantageous for transferring genomic information between species [3-5,72]. This raises the prospect that evolutionary genomic studies of cyprinid fishes are accelerated by the interspecific exchange of information and by complementary studies between species.

## Conclusions

We constructed a highly dense linkage map of gudgeons (*Gnathopogon*) using RAD-seq. This map covers a majority of the genome, and the number of linkage groups is consistent with the haploid chromosome number of *Gnathopogon*. Sex-specific departure from a Mendelian inheritance pattern was identified in a linkage group. Synteny and collinearity are highly conserved between *Gnathopogon* and the traditional model organism zebrafish. We inferred that extensive interchromosomal rearrangements are not common between *Gnathopogon* and zebrafish, but intrachromosomal rearrangements have occurred. This linkage map clarifies the genetic architecture underlying the morphological diversification of *Gnathopogon* using the future QTL analysis. The transfer of genomic information from zebrafish to *Gnathopogon*, enabled by their conserved synteny and collinearity, is also useful for screening candidate genes responsible for the traits of interest.

## Competing interests

The authors declare that they have no competing interests.

## Authors’ contributions

TK and NO initiated the research project on trophic polymorphism in *Gnathopogon*. RK, TK, KW, and NO conceived and designed the experiments. RK, TK, and HK performed the experiments. RK conducted data analysis. RK and TK wrote the paper. All authors read and approved the final manuscript.

## Supplementary Material

Additional file 1 List of RAD stack sequences, including consensus sequences of RAD loci. Click here for file

Additional file 2: Figure S1 A detailed linkage map of the interspecific cross between *Gnathopogon caerulescens* and *Gnathopogon elongatus*. The lengths of the linkage groups are based on Kosambi cM. Click here for file

Additional file 3: Figure S2 Linkage maps of LG3, LG3M, and LG3D, and extent of deviation from Mendelian segregation in LG3D. (A) Linkage maps of LG3, LG3M, and LG3D. The map distance estimates are in Kosambi cM. The homology of loci between linkage groups is indicated with a line. (B) Degree of deviation from the expected 1:2:1 segregation along LG3D. The *x*-axis represents the position on LG3D; *y*-axis represents *χ*^2^-test *P*-values for distorted segregation. The dotted line represent *α* = 0.001. Click here for file

Additional file 4: Figure S3 Comparison of syntenic pairs of a linkage group of *Gnathopogon* and a chromosome of zebrafish. Each dot represents the position of a homologous locus. The *x*-axis is proportional to physical length; the *y*-axis is proportional to Kosambi cM. Click here for file
