# Supplementary material for: A RAD-based linkage map and comparative genomics in the gudgeons (genus Gnathopogon, Cyprinidae)
Source: BMC Genomics. 2013 Jan 16;14:32. doi: 10.1186/1471-2164-14-32 (PMC3583795; doi:10.1186/1471-2164-14-32)
Supplement: Additional file 2: Figure S1 — A detailed linkage map of the interspecific cross between Gnathopogon caerulescens and Gnathopogon elongatus. The lengths of the linkage groups are based on Kosambi cM. [file 1471-2164-14-32-S2.pdf]

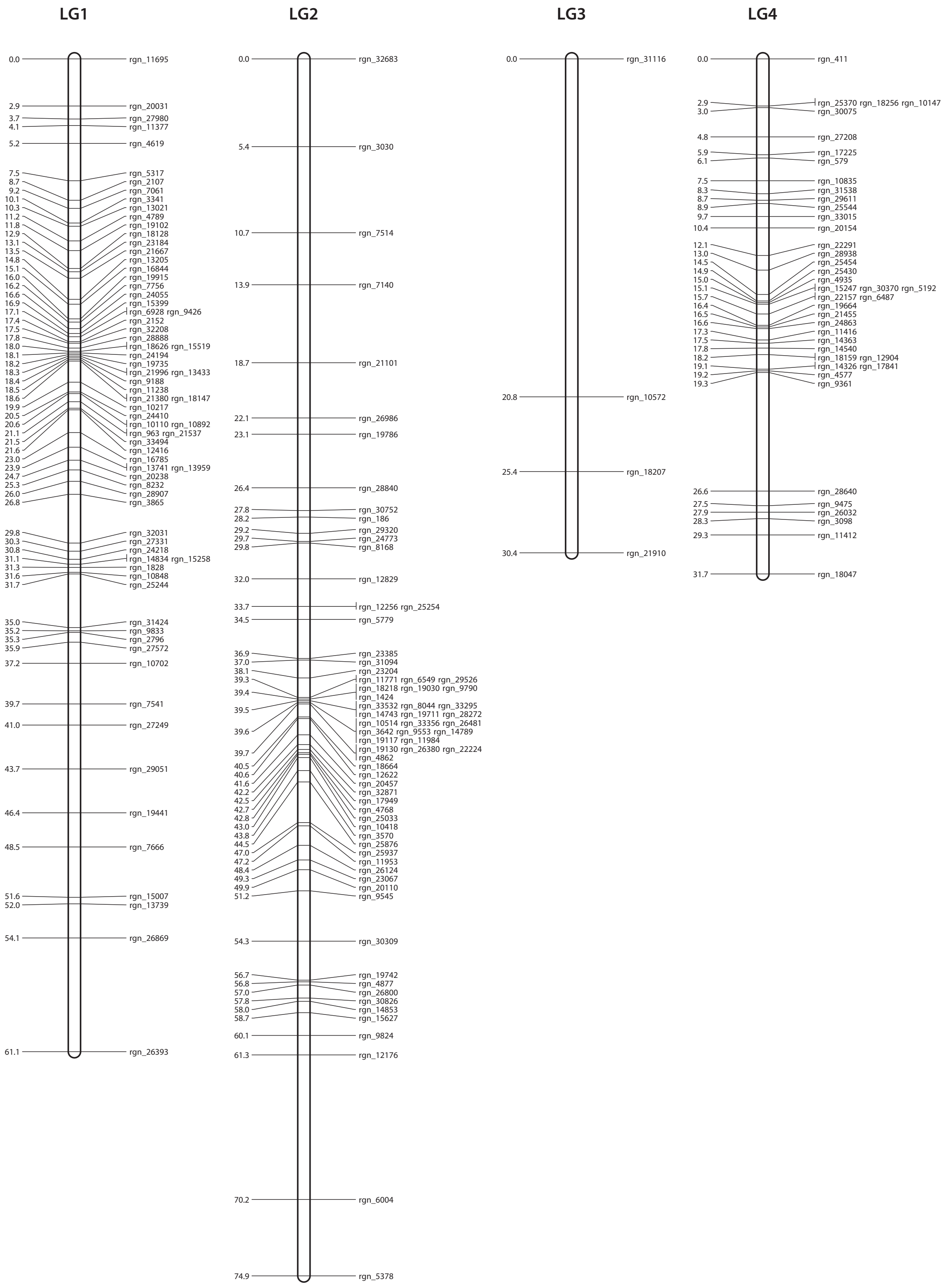

Figure S1 A detailed linkage map of the interspecific cross between *Gnathopogon caerulescens* and *Gnathopogon elongatus*. The lengths of the linkage groups are based on Kosambi cM.

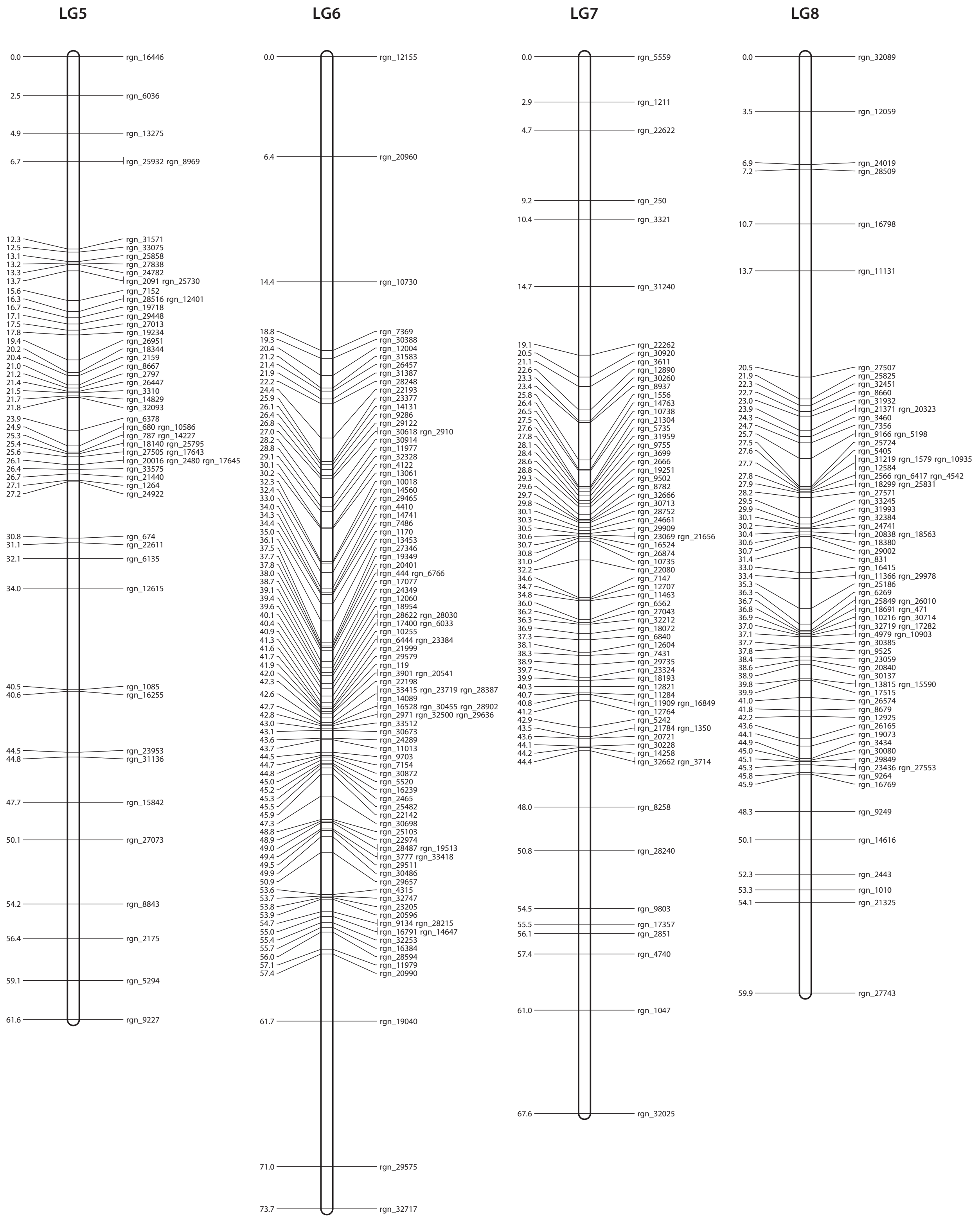

Figure S1 (continued)

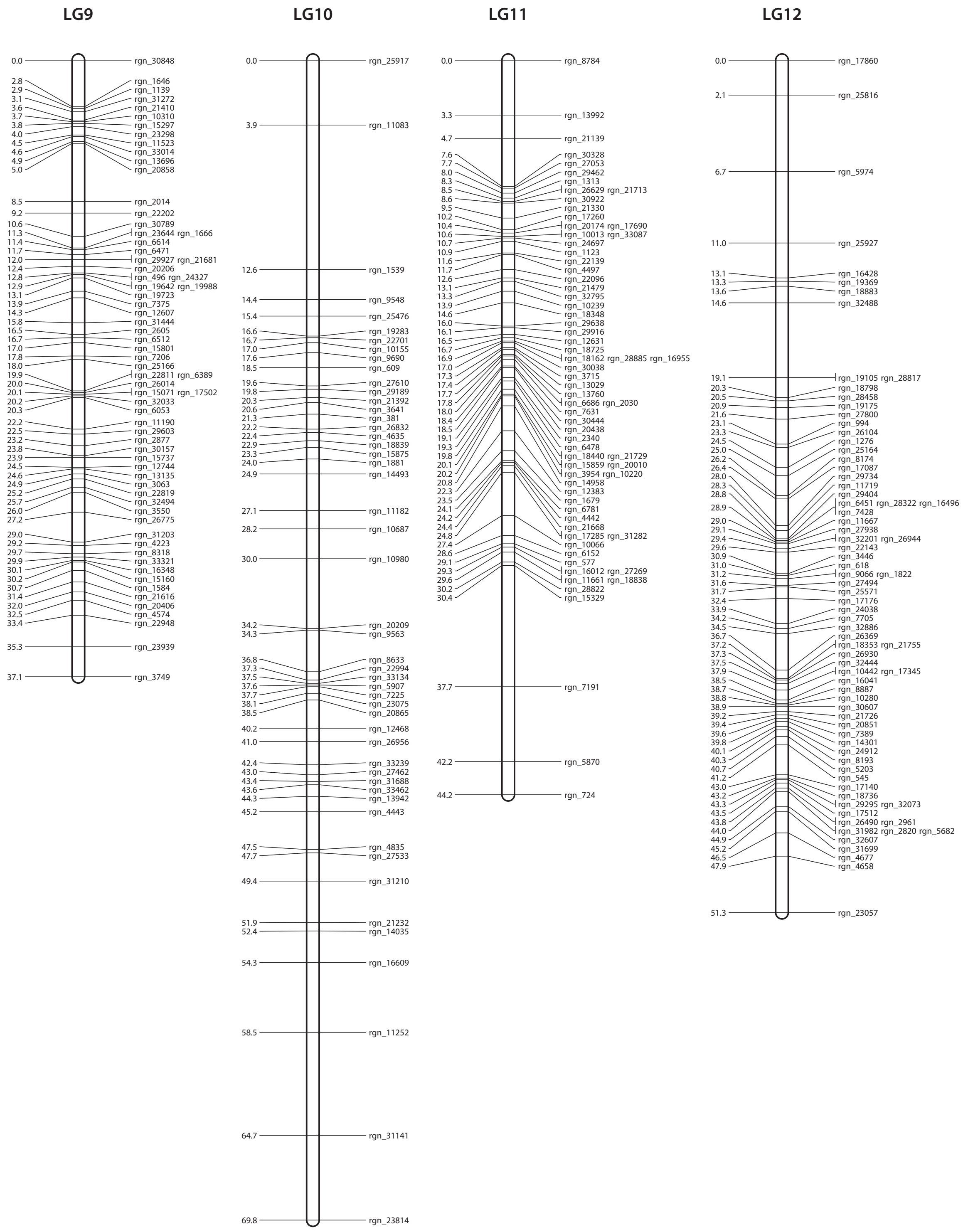

Figure S1 (continued)

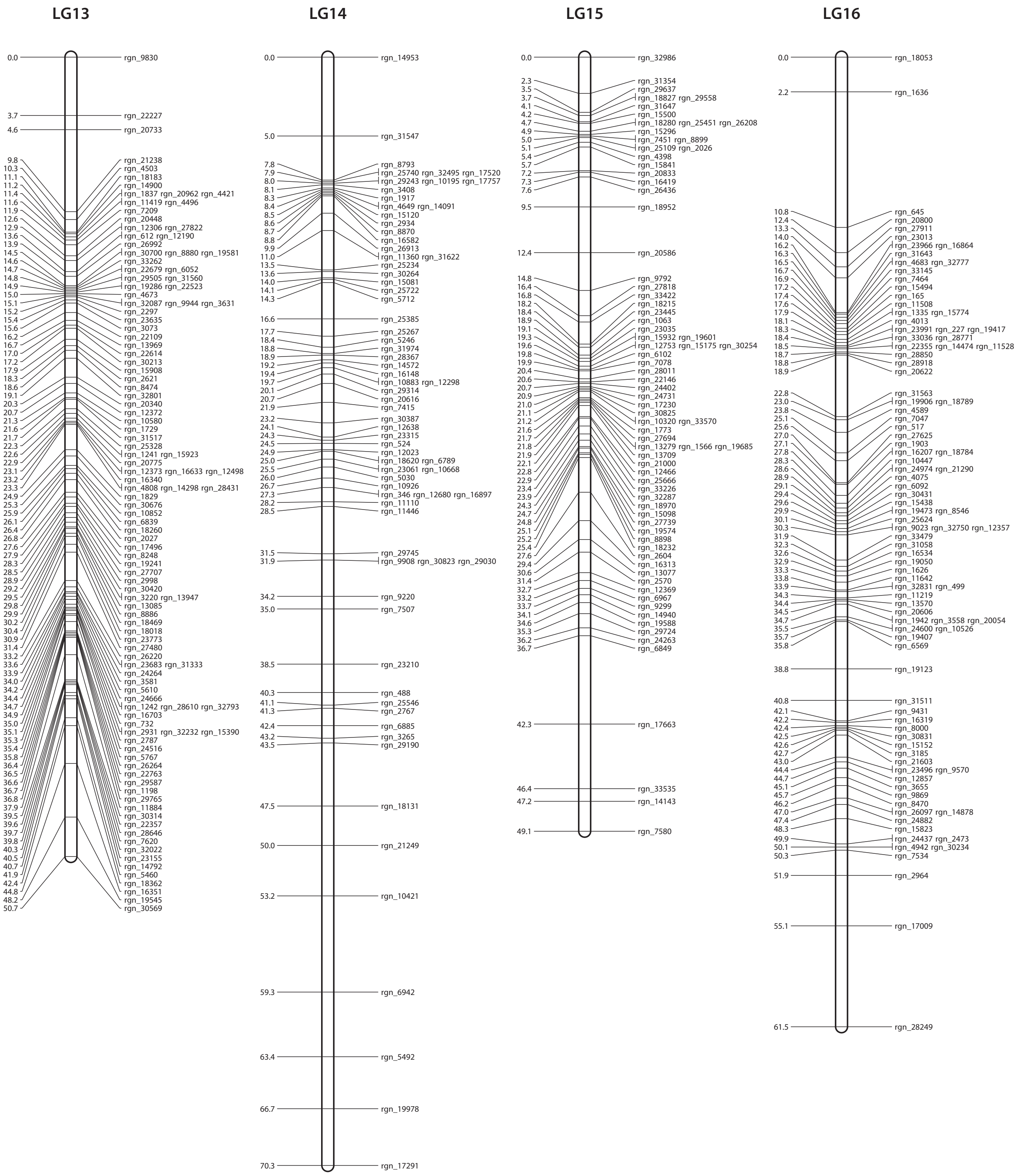

Figure S1 (continued)

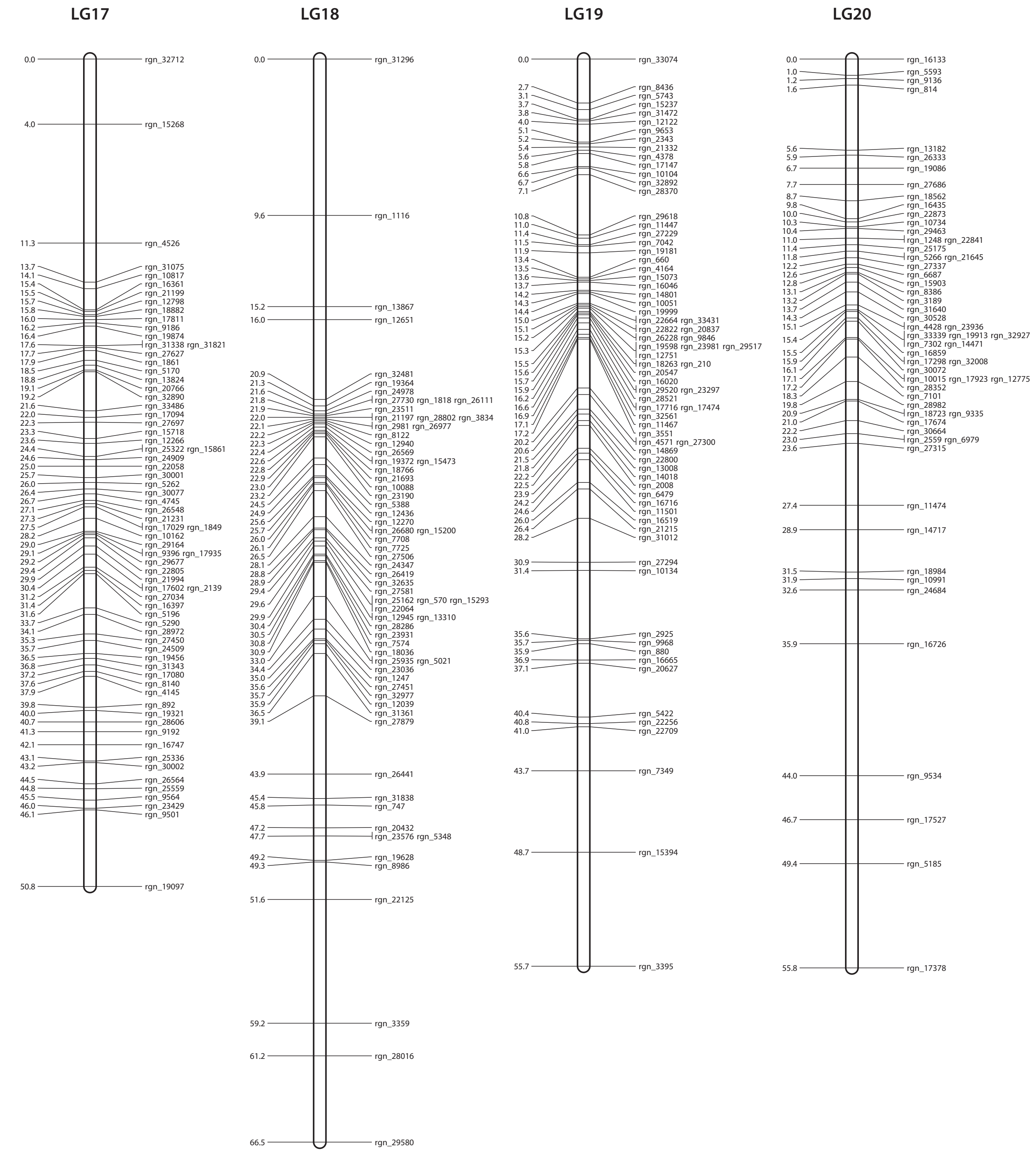

Figure S1 (continued)

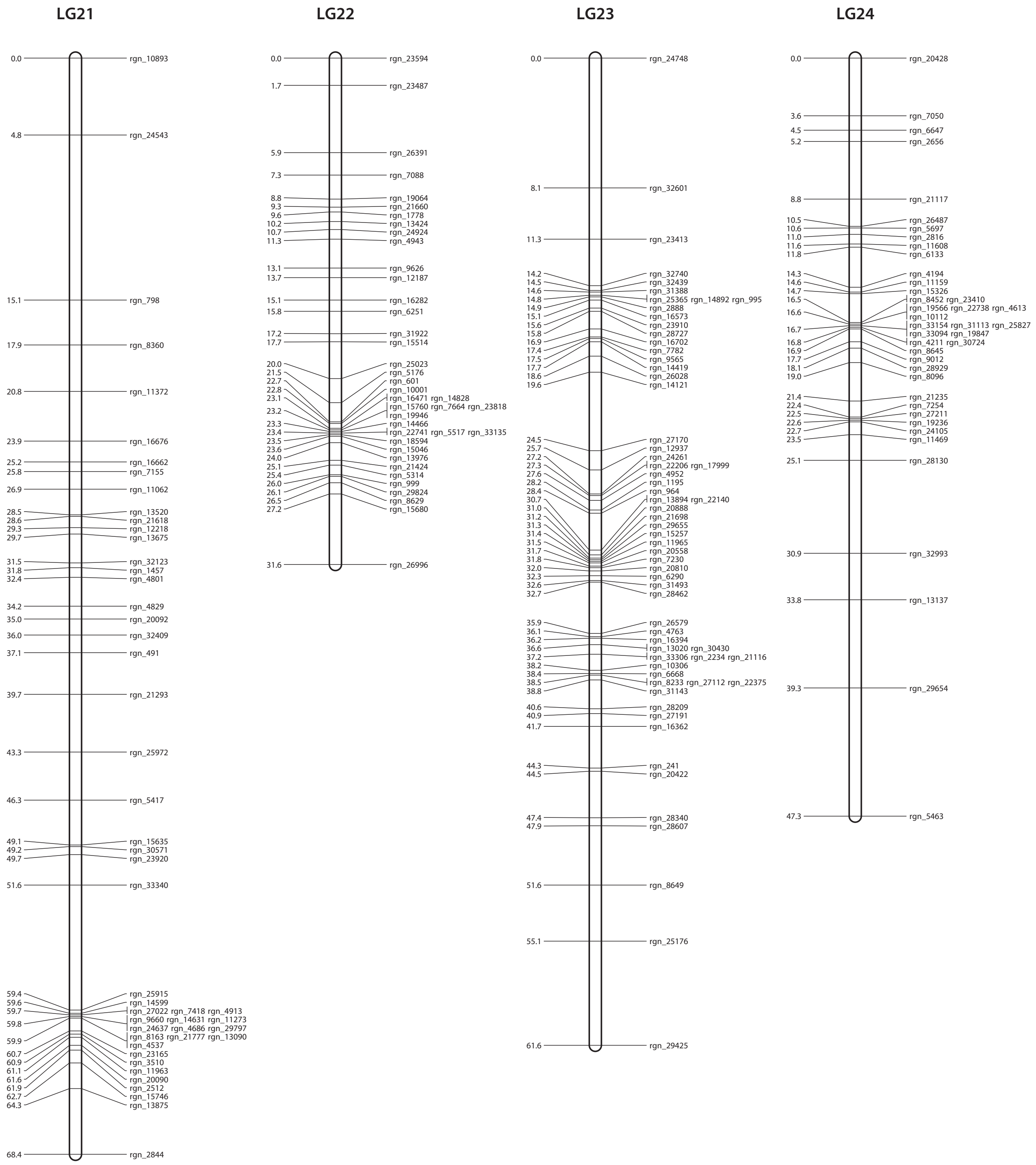

Figure S1 (continued)

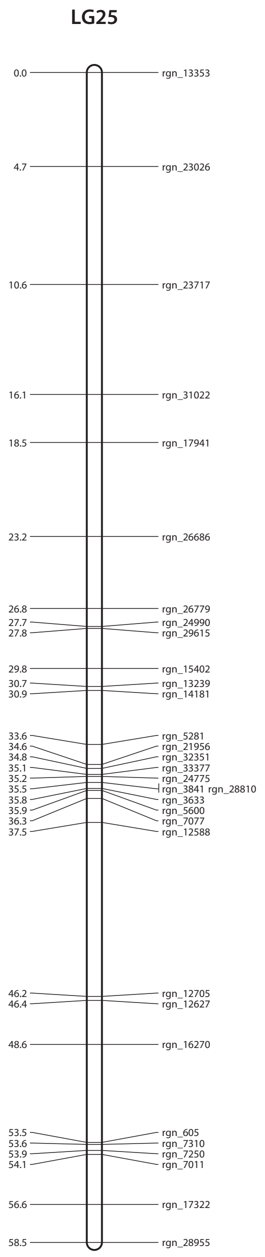

Figure S1 (continued)
