# Supplementary material for: A RAD-based linkage map and comparative genomics in the gudgeons (genus Gnathopogon, Cyprinidae)
Source: BMC Genomics. 2013 Jan 16;14:32. doi: 10.1186/1471-2164-14-32 (PMC3583795; doi:10.1186/1471-2164-14-32)
Supplement: Additional file 3: Figure S2 — Linkage maps of LG3, LG3M, and LG3D, and extent of deviation from Mendelian segregation in LG3D. (A) Linkage maps of LG3, LG3M, and LG3D. The map distance estimates are in Kosambi cM. The homology of loci between linkage groups is indicated with a line. (B) Degree of deviation from the expected 1:2:1 segregation along LG3D. The x-axis represents the position on LG3D; y-axis represents χ2-test P-values for distorted segregation. The dotted line represent α = 0.001. [file 1471-2164-14-32-S3.pdf]

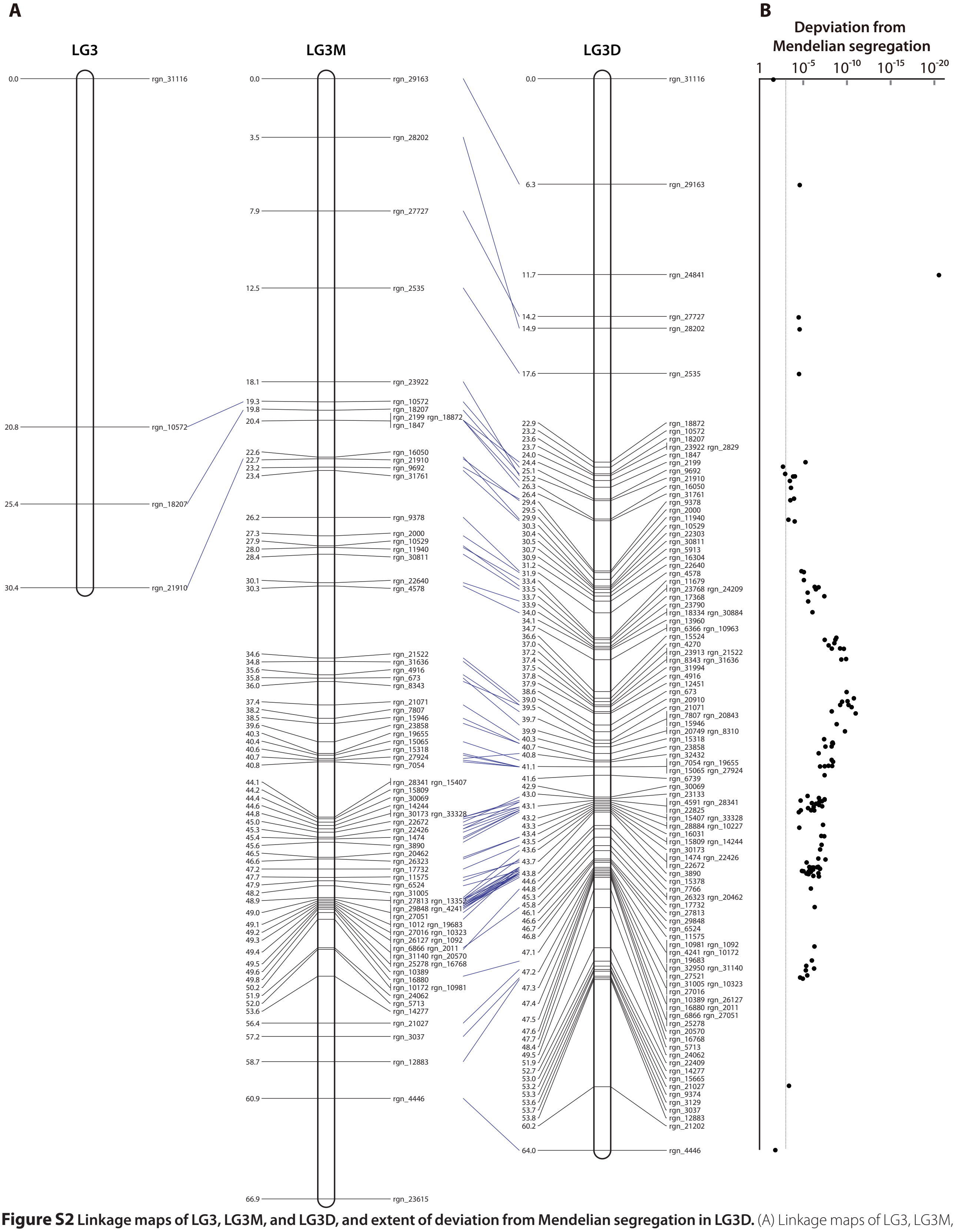

Figure S2 Linkage maps of LG3, LG3M, and LG3D, and extent of deviation from Mendelian segregation in LG3D. (A) Linkage maps of LG3, LG3M, and LG3D. The map distance estimates are in Kosambi cM. The homology of loci between linkage groups is indicated with a line. (B) Degree of deviation from the expected 1:2:1 segregation along LG3D. The *x*-axis represents the position in LG3M; *y*-axis represents χ^2-test *P*-values for distorted segregation. The dotted line represent $\alpha = 0.001$.
