# Supplementary material for: A RAD-based linkage map and comparative genomics in the gudgeons (genus Gnathopogon, Cyprinidae)
Source: BMC Genomics. 2013 Jan 16;14:32. doi: 10.1186/1471-2164-14-32 (PMC3583795; doi:10.1186/1471-2164-14-32)
Supplement: Additional file 4: Figure S3 — Comparison of syntenic pairs of a linkage group of Gnathopogon and a chromosome of zebrafish. Each dot represents the position of a homologous locus. The x-axis is proportional to physical length; the y-axis is proportional to Kosambi cM. [file 1471-2164-14-32-S4.pdf]

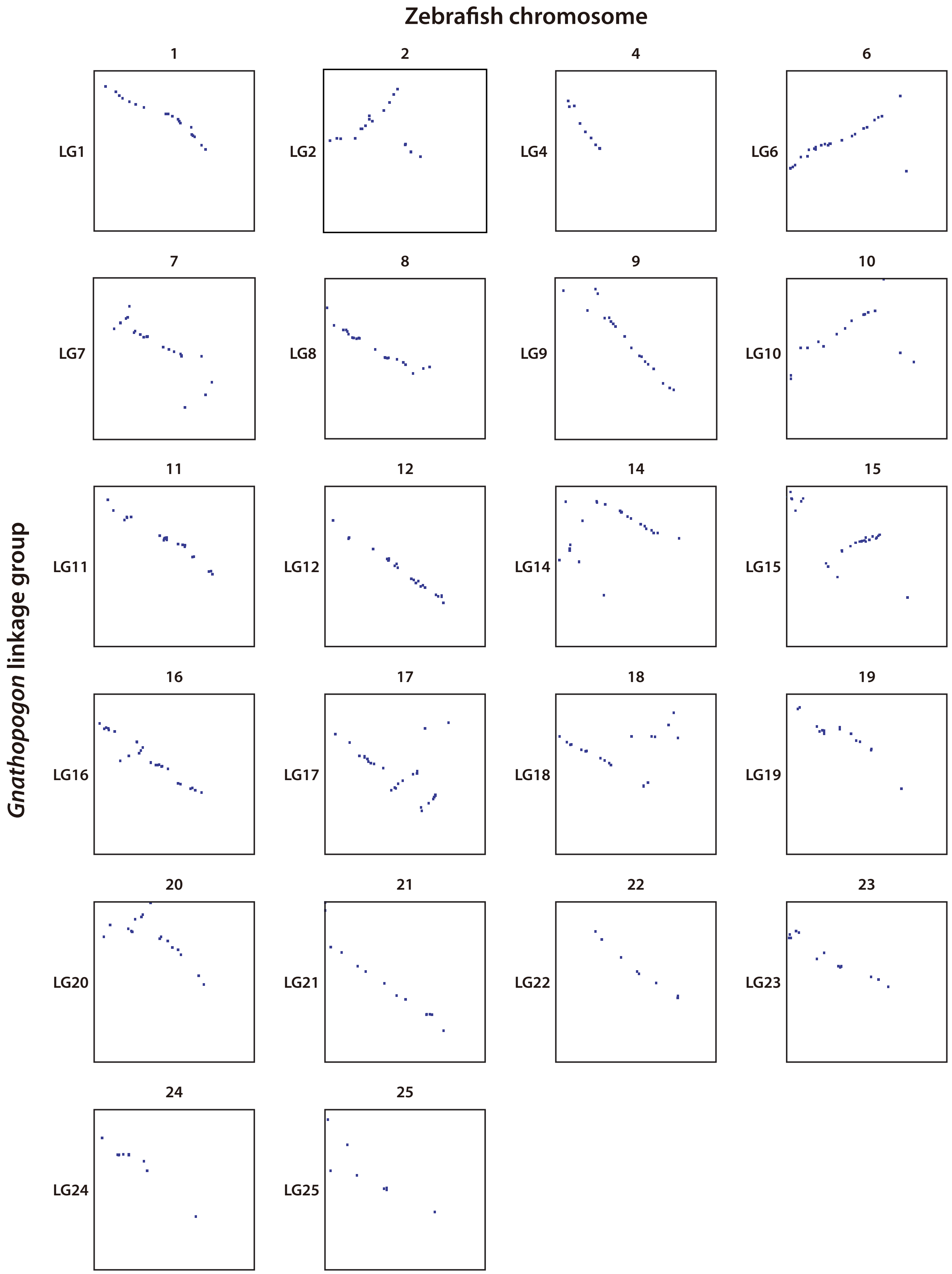

Figure S3 Comparison of syntenic pairs of chromosomes of *Gnathopogon* and zebrafish. Each dot represents the position of a homologous locus. The *x*-axis is proportional to physical length; the *y*-axis is proportional to Kosambi cM.
